# Multicolor lineage tracing reveals clonal architecture and dynamics in colon cancer

**DOI:** 10.1038/s41467-017-00976-9

**Published:** 2017-11-10

**Authors:** Sebastian Lamprecht, Eva Marina Schmidt, Cristina Blaj, Heiko Hermeking, Andreas Jung, Thomas Kirchner, David Horst

**Affiliations:** 1 Pathologisches Institut, Ludwig-Maximilians-Universität München, Thalkirchner Str. 36, 80337 München, Germany; 2 German Cancer Consortium (DKTK), 69120 Heidelberg, Germany; 3 German Cancer Research Center (DKFZ), 69120 Heidelberg, Germany

## Abstract

Colon cancers are composed of phenotypically heterogeneous tumor cell subpopulations with variable expression of putative stem cell and differentiation antigens. While in normal colonic mucosa, clonal repopulation occurs along differentiation gradients from crypt base toward crypt apex, the clonal architecture of colon cancer and the relevance of tumor cell subpopulations for clonal outgrowth are poorly understood. Using a multicolor lineage tracing approach in colon cancer xenografts that reflect primary colon cancer architecture, we here demonstrate that clonal outgrowth is mainly driven by tumor cells located at the leading tumor edge with clonal axis formation toward the tumor center. While our findings are compatible with lineage outgrowth in a cancer stem cell model, they suggest that in colorectal cancer tumor cell position may be more important for clonal outgrowth than tumor cell phenotype.

## Abstract (web summary)

The clonal architecture of colon cancer and the relevance of tumor cell subpopulations for clonal outgrowth are poorly understood. Here, the authors describe the clonal architecture and dynamics in human colon cancer by using a multicolor lineage tracing approach in colon cancer xenografts.

## Introduction

Colorectal cancer derives from normal colonic mucosa by stepwise accumulation of mutations that transform epithelial cells into invasively growing tumors. Normal colonic mucosa is quite simply organized, basically as a sheet of epithelial cells with infoldings forming stereotypical crypts. These are continuously clonally repopulated by stem cells from the crypt base with maturation toward the crypt apex^1^. In contrast to normal colonic mucosa, the architecture of colon cancer is much less understood since these tumors form masses with varying degrees of morphologically disarrayed epithelial glands^2^. However, colon cancers do not appear to be completely unorganized. Gradients from less differentiated tumor cells at the leading tumor edge to glandular differentiated tumor cells in the tumor center can be observed in many cases, and mimic the polarity of normal colonic crypts to varying extent^3, 4^. However, in contrast to normal colonic crypts, such gradients in colon cancer are not situated within stereotypical morphological units and some colon cancers even lack differentiation gradients. Therefore, the relevance of phenotypically distinct tumor cells for tumor growth and the resulting colon cancer architecture remains incompletely understood.

Colon cancer cell subpopulations with distinct phenotypes and degrees of differentiation may have different functions. Most prominently, tumor initiating potential has been attributed to colon cancer cells with high WNT and MAPK signaling activity^5, 6^. In well-differentiated colon cancers, such tumor cells are frequently located close to the infiltrative tumor edge, leading to the hypothesis that colon cancer stem cells reside at this location^7^. However, defining colon cancer stem cells through tumor-initiating potential, the current gold standard, may have certain limitations and cannot always be generalized^8, 9^. Moreover, it has been questioned whether the position of a cell within the cellular hierarchy of a growing tumor is adequately reflected by tumor-initiating potential^10^. Therefore, from these data, the role of distinct tumor cell phenotypes for the dynamics of clonal expansion in colon cancer has remained unclear.

Recently, lineage tracing tools have been developed that allow assessing cell fate restriction in temporal order, and were used to define clonal dynamics in genetically engineered mouse tumor models^11, 12^. Moreover, current studies demonstrated clonal outgrowth from colon cancer cells with high MAPK activity or expression of the WNT target gene *LGR5*, and thus provided direct evidence for a cellular hierarchy emanating from these tumor cell subsets in vivo^13, 14^. Here we used a phenotype-independent multicolor lineage tracing system for quantitative cell fate analysis in colon cancer xenografts. We demonstrate that clonal expansion starts at the leading tumor edge, where tumor cells compete for outgrowth toward the tumor center, where clones may be lost due to tumor necrosis. Our findings suggest that tumor cell position may be more important for lineage persistence than tumor cell phenotype. This new concept may have implications for the cancer stem cell hypothesis and for the design of therapeutic strategies.

## Results

### Tumor cell differentiation gradients in colon cancer

**Fig. 1:**
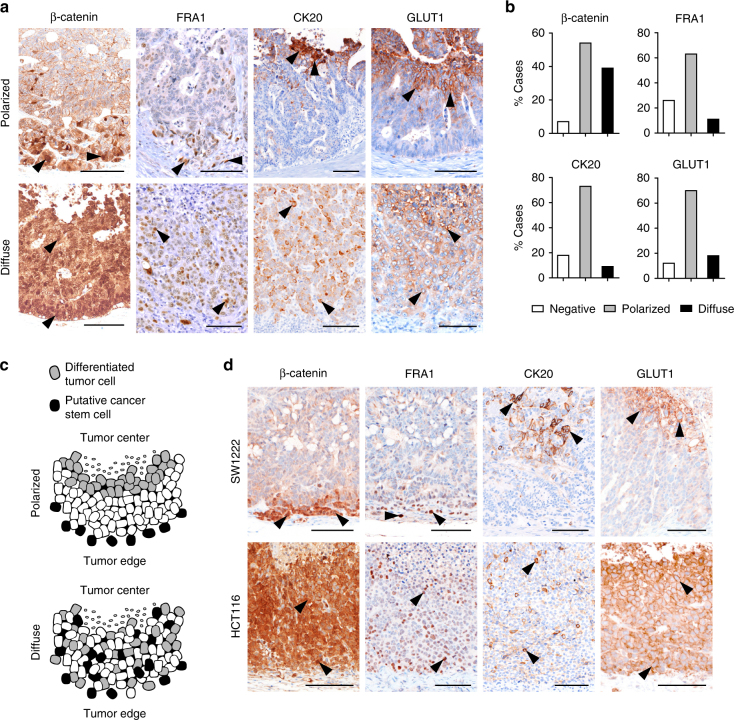
Varying degrees of differentiation gradients in colon cancer. **a** Immunohistochemistry for indicated proteins in representative primary colon cancers demonstrates examples of polarized or diffuse marker distribution. **b** Frequencies of observed marker distributions in colon cancer (*n* = 92). **c** Schematic model for colon cancers with polarized or diffuse marker distribution. **d** Immunohistochemistry for indicated proteins in SW1222- and HCT116-derived xenografts demonstrates polarized or diffuse marker expression, respectively. All micrographs show tumors from leading tumor edge (image bottom) to tumor center or central tumor necrosis (image top). Arrowheads indicate positively stained tumor cells. Scale bars, 100 µm

First, we assessed primary colon cancers for the expression of nuclear β-catenin and FRA1 as surrogate markers for high WNT and MAPK signaling^15, 16^ that were previously linked to tumor-initiating potential and colon cancer stem cells. In addition, we determined expression of CK20 and GLUT1 that in contrast indicated epithelial cell differentiation and hypoxia, respectively^17, 18^. Many colon cancers showed increased nuclear β-catenin and FRA1 expression in tumor cells located at the infiltrative tumor edge, whereas CK20 and GLUT1 were most strongly expressed in the tumor center, often close to necrotic areas, suggesting a polarity with differentiation gradients directed from the tumor edge toward the tumor center (Fig. 1a, b and Supplementary Fig. 1). However, a substantial number of colon cancers did not show definite intratumoral differentiation gradients, since they either expressed these markers more diffusely throughout the tumor, or were negative for individual markers (Fig. 1a, b and Supplementary Fig. 1). These findings suggested that colon cancers may be categorized into tumors with polarized or more diffuse expression of differentiation antigens and markers that were previously related to colon cancer stem cells (Fig. 1c).

Next, we characterized a collection of colon cancer xenografts and found that SW1222-derived tumors showed a polarized distribution of nuclear β-catenin, FRA1, CK20, and GLUT1, while HCT116 colon cancer xenografts showed more diffuse marker expression and lack of differentiation gradients (Fig. 1d). We therefore used xenografts of these two cell lines as model tumors for the typical spectrum of presence or absence of differentiation gradients that is observed in primary colon cancers.

### Multicolor lineage tracing in colon cancer in vivo

**Fig. 2:**
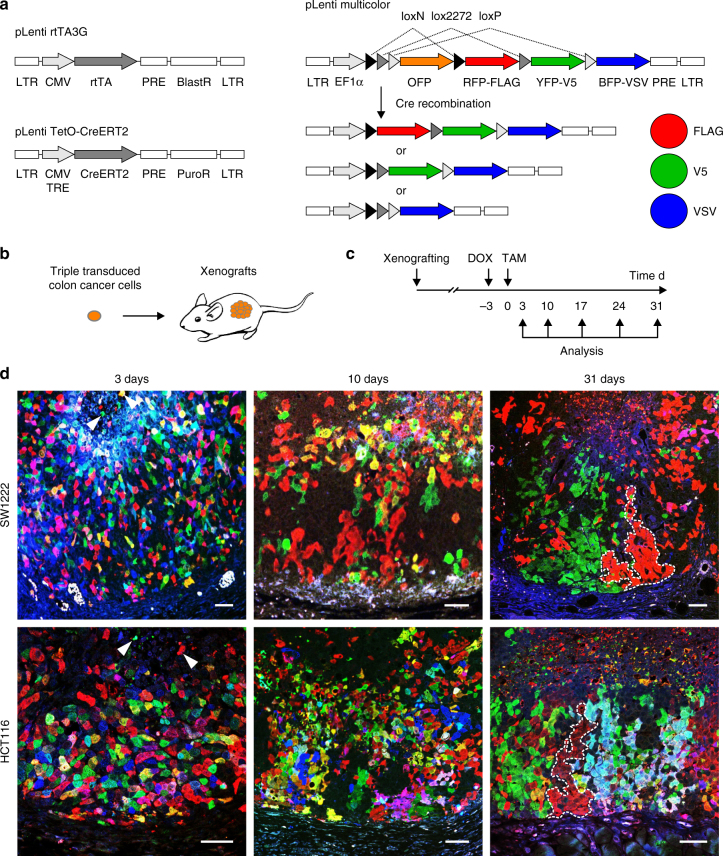
Multicolor lineage tracing in colon cancer xenografts. **a** Lentiviral vectors for expression of rtTA (pLenti rtTA3G), doxycycline-dependent CreERT2 (pLenti TetO-CreERT2), and the Cre-responsive multicolor transgene (pLenti Multicolor). Upon Cre-recombination, transgene elements flanked by loxN, lox2272, or loxP sites will be removed at random, causing an irreversible switch from expression of orange (OFP) to tagged red (RFP-FLAG), yellow (YFP-V5), or blue fluorescent proteins (BFP-VSV), respectively. LTR long terminal repeat, TRE tetracycline response element, BlastR/PuroR blasticidin and puromycin resistance genes. PRE posttranscriptional regulatory element. **b** Triple transduced colon cancer cells were xenografted into NOD/SCID mice. **c** Schedule for doxycycline (DOX) and tamoxifen (TAM) treatment, and tumor harvest after xenografting. **d** Confocal immune fluorescence for RFP-FLAG (red), YFP-V5 (green), and BFP-VSV (blue) in xenografts at indicated time points after tamoxifen-induced multicolor labeling. Individual clones at 31 days are indicated by dotted lines. Fluorescent images show xenograft tumors from leading tumor edge (image bottom) to central tumor necrosis (image top). Arrowheads indicate loss of tumor cell clones into tumor necrosis. Scale bars, 50 µm

In order to visualize lineage outgrowth in colon cancer, we developed a lentiviral Cre recombinase sensitive reporter system that allowed stochastic expression of different fluorescent colors in individual tumor cells. Our system consists of three lentiviral vectors, two of which mediate doxycycline-inducible expression of an estrogen receptor-Cre fusion protein (pLenti rtTA3G and pLenti TetO-CreERT2). Upon Cre recombination, the third vector, similar to a Brainbow transgene^19^, randomly switches from expression of orange to either tagged red, yellow, or blue fluorescence proteins (pLenti Multicolor, Fig. 2a). This doxycycline and tamoxifen-controlled design was completely devoid of unwanted background recombination. We then transduced all three vectors into SW1222 and HCT116 colon cancer cells, expanded single cell clones, and xenografted them into immune compromised NOD/SCID mice (Fig. 2b). After xenograft growth, we induced recombination by doxycycline and tamoxifen treatment and analyzed clonal outgrowth over time (Fig. 2c). Three days after induction of recombination, individual or small clusters of colon cancer cells were randomly labeled by different fluorescent colors in a mosaic pattern throughout SW1222 and HCT116 xenograft tumors. Of note, integration of multiple vectors resulted in various mixed fluorescent colors (Supplementary Fig. 2). Interestingly, at this early time point after recombination, we already observed loss of few color-labeled clones into the central tumor necrosis (Fig. 2d). Ten days after recombination, single color clones had increased in size, while at 31 days after recombination, large stripe- and wedge-like shaped clones extended from the tumor edge to the necrotic tumor center (Fig. 2d and Supplementary Fig. 3). Inducible multicolor tracing thus allowed us to monitor clonal outgrowth within human colon cancer in vivo, and suggested clonal expansion along axes from the tumor edge toward the tumor center.

### Clone characteristics in colon cancer

**Fig. 3:**
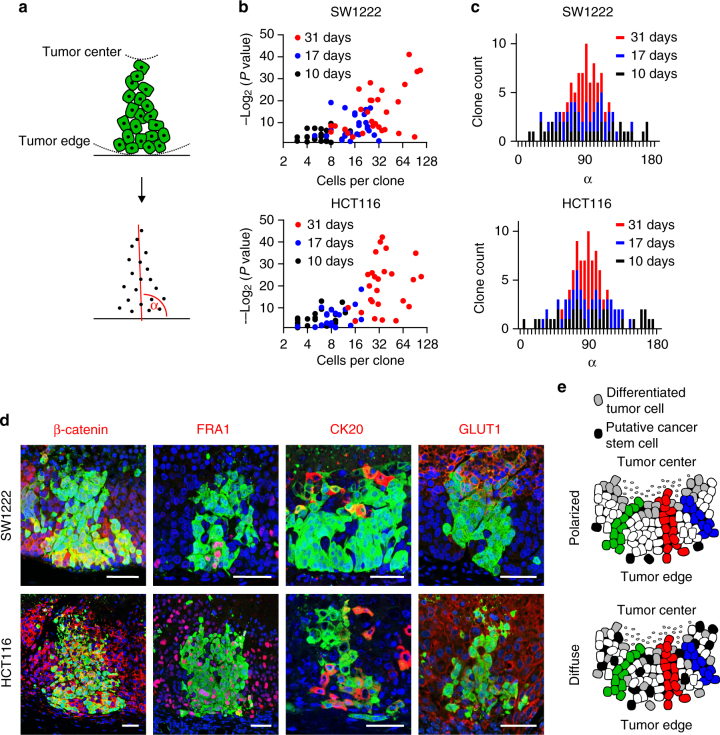
Shape and axis formation of colon cancer subclones. **a** Schematic illustration of clonal analysis. Positions of cells in individual clones relative to leading tumor edge and tumor center were determined. For each clone, a line of best fit for cell positions was calculated by linear regression, yielding a clonal axis. *α* indicates the angle of the clonal axis relative to the leading tumor edge. **b** Significance (*P* values of linear regression) of linear alignment of cells in individual clones of different sizes at indicated time points after multicolor labeling. **c** Angles (*α*) of clonal axes relative to the leading tumor edge at indicated time points after multicolor labeling. **d** Confocal images show positions of indicated stem cell and differentiation antigens (red) in individual clones (green) of colon cancer xenografts. Fluorescent images show xenograft tumors from leading tumor edge (image bottom) to central tumor necrosis (image top). Scale bars, 50 µm. **e** Schematic model suggesting identical clonal outgrowth in colon cancers with polarized and diffuse marker distribution

To characterize the shape and architecture of colon cancer subclones in more detail, we determined the coordinates of coherent tumor cells with identical colors relative to perpendicular linear axes from the tumor edge to the tumor center (Fig. 3a). An adapted model for linear regression analyses revealed that 31 days after recombination most clones had expanded in a linear manner in SW1222 and HCT116 xenografts, while we observed this with less significance at earlier time points (Fig. 3b). Moreover, when we determined the angles (*α*) of lines fitted to clones by linear regression relative to tangents to the leading tumor edge (Fig. 3a), these predominantly centered around 90° in tumors of both cell lines (Fig. 3c). In addition, we then performed BrdU tracing experiments and found that within 6 or 7 days after a single BrdU pulse, the label progressed from the tumor edge toward the tumor center (Supplementary Fig. 4). Collectively, these findings provided evidence of a non-random linear expansion of tumor cell clones, perpendicular to the leading tumor edge and directed toward the tumor center.

With these findings in mind, we analyzed the distribution of nuclear β-catenin, FRA1, CK20, and GLUT1 within individual clones. As expected for SW1222-derived xenografts, nuclear β-catenin and FRA1 marked tumor cells predominantly at the leading tumor edge, while CK20 and GLUT1 marked cells close to the necrotic tumor center within these clones, indicating clonal axis formation along the polarity of the centripetal differentiation axis in these tumors (Fig. 3d). Importantly however, since clonal axes in HCT116 colon cancer xenografts also were perpendicular to the leading tumor edge, and all four markers were diffusely expressed in individual clones of these tumors, this indicated that clonal axis formation does not generally parallel or depend on differentiation gradients (Fig. 3d, e). These findings suggested limited influence of differentiation gradients on clonal architecture and outgrowth in colon cancer.

### Clonal dynamics in colon cancer

**Fig. 4:**
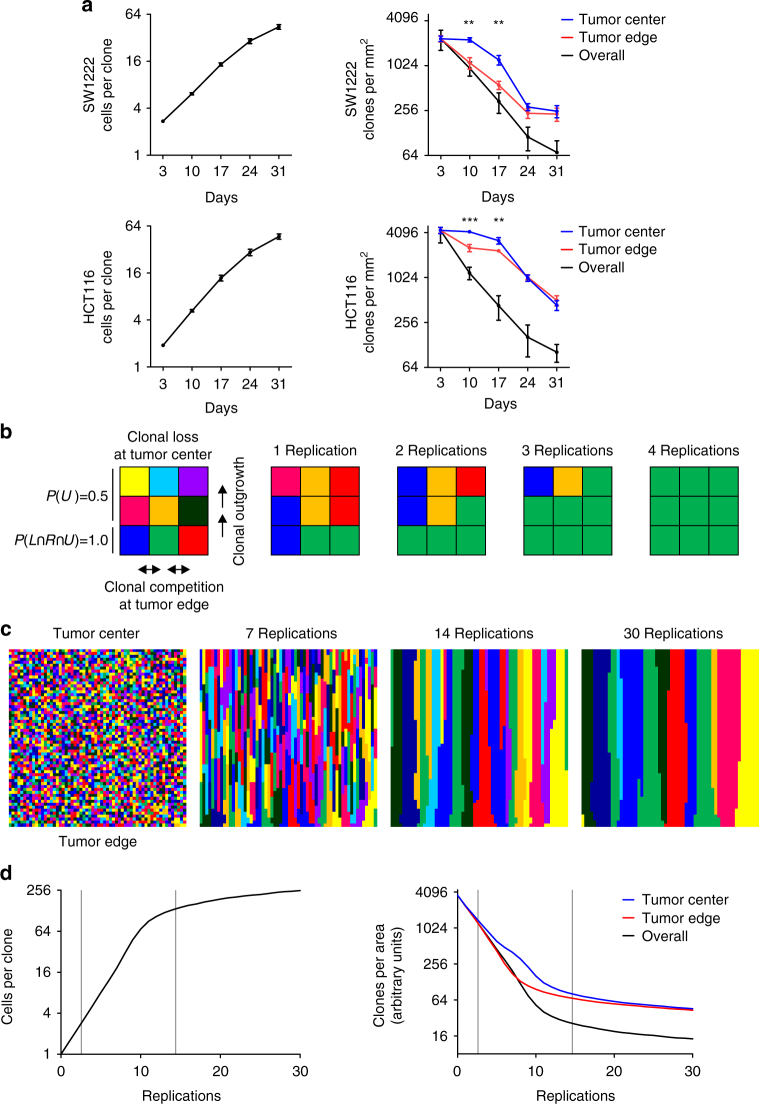
Analysis and simulation of clonal dynamics in colon cancer. **a** Clone sizes (left panels) and clones per area (right panels), as determined by analysis of confocal fluorescence images of SW1222 and HCT116 colon cancer xenografts at different time points after multicolor labeling. Clones per area were measured overall and separately in tumor thirds close to the tumor edge and close to the tumor center, as indicated. Data are mean and error bars indicate s.e.m. (left panels) and s.d. (right panels). ***P < 0.001 and **P < 0.01 indicate differences between tumor edge and tumor center by two-sided *t*-test. **b** Two-dimensional simulation model for clonal dynamics. Bottom row simulates tumor cells at the tumor edge and top row simulates tumor cells neighboring central tumor necrosis. At the tumor edge, cells divide during each simulated replication cycle and probabilities for cell expansion upwards *P*(*U*), to the left *P*(*L*) or to the right *P*(*R*) are equal. In other positions, cells divide upwards at decreased frequency with *P*(*U*) = 0.5. One possible outcome after four replication cycles is illustrated. **c** Scaling this model to 60 × 60 cells simulates clones expanding from tumor edge to tumor center. One possible outcome after 30 replication cycles is shown. **d** Average clone sizes (left panel) and clones per area (right panel) from 100 independent simulations. Clones per area are given overall, and in thirds of the model close to tumor edge and tumor center, respectively. Gray lines approximately deliminate simulation segments fitting our in vivo data

To further learn about clonal dynamics in colon cancer xenografts, we analyzed clone sizes and clonal density after multicolor labeling over time. Three days after recombination, clones were composed of two–three cells in average. Clone sizes then increased exponentially until 17 days with subsequently slightly slowed growth rates (Fig. 4a). Accordingly, clonal density, i.e., the number of clones per area, decreased over time. Importantly, when comparing clonal density at the tumor edge and in the tumor center, we observed a significantly earlier decrease in clonal density at the leading tumor edge, most obvious at 10 days and 17 days after recombination in both SW1222 and HCT116 colon cancer xenografts (Fig. 4a). Also, over time the average clonal width at the leading tumor edge increased when adjusted to increases in tumor circumference (Supplementary Fig. 5). Together with the observation that clones could be lost due to central tumor necrosis, these findings suggested a clonal competition at the leading tumor edge with subsequent clonal outgrowth toward the tumor center. Of note, when we analyzed individual clones 31 days after recombination for cancer hot spot mutations, no mutational differences were observed. This indicated that clonal outgrowth and competition likely occurred in the absence of overt changes in driver mutation profiles (Supplementary Table 1).

Finally, we inferred a two-dimensional spatial simulation model for clonal dynamics in colon cancer (Supplementary Data 1), implementing few rules only that we derived from our in vivo observations (Fig. 4b). First, clones may only be lost into the tumor center or into central tumor necrosis, represented by the upper border of our square model. Second, clonal competition by lateral clone expansion may only occur at the leading tumor edge, represented by the lower border of the model. Third, based on the measurements of proliferation by Ki67 in primary colon cancers (*n* = 92) and xenograft tumors (Supplementary Fig. 6), growth rates in our model were slowed to 0.5 in central tumor areas relative to the leading tumor edge. This model, when composed of few “cells” only, illustrated rapid loss of individual tumor cells and a drift toward mono-clonality (Fig. 4b). In larger scale, linear expansion of tumor cell clones from the leading tumor edge toward the tumor center were seen with widening of some clones and inevitable loss of those that lost contact to the leading edge, causing a continuous drift toward oligoclonality, and well fitting our in vivo findings (Fig. 4c). Also, the dynamics of gains in clone size and loss in clone density over time quantitatively matched our observations in colon cancer xenografts (Fig. 4d). Importantly, this also included an earlier decrease of clonal density at the leading tumor edge compared to the tumor center, which was due to the implemented restriction of clonal competition to the leading tumor edge. Collectively, this model corroborated the idea that the in vivo observed clonal outgrowth from the leading tumor edge toward the tumor center may be based on few rather positional characteristics of colon cancer cells while differentiation gradients and polarity may be of less importance.

## Discussion

In this study, we implemented a quantitative lineage tracing strategy to gain insights into the clonal expansion dynamics of individual tumor cells within growing colon cancer in vivo. We used cell line-derived colon cancer xenografts as model tumors to reproduce the architecture, cellular composition, and differentiation of primary human colon cancers^4^. Our data suggest that colon cancer cells at the leading tumor edge compete for clonal outgrowth, which is directed toward the tumor center. In tumors with polarized differentiation gradients, this clonal expansion may coincide with tumor cell differentiation. These findings are in agreement with recent data demonstrating clonal outgrowth from tumor cells with high MAPK pathway activity or high expression of the WNT target gene *LGR5* at the leading tumor edge^13, 14^. In this case, linear expansion of tumor cell subclones may be well compatible with lineage outgrowth from phenotypically defined colon cancer stem cells^10^. Despite a distorted architecture, clonal outgrowth and differentiation in colon cancer therefore can be reminiscent of normal colonic mucosa, where stem cells at the crypt base compete for clonal repopulation of individual crypts^1^.

However, colon cancer xenografts lacking differentiation gradients, and thus more diffusely distributed expression of putative stem cell and differentiation antigens, unexpectedly showed the same pattern of clonal expansion from tumor edge to center. Therefore, clonal fitness and positive clonal selection rather appear to depend on positioning of tumor cells at the leading tumor edge than on tumor cell differentiation. Indeed, when considering broad expression of some putative cancer stem cell antigens in colon cancers, it may be difficult to imagine how a cancer stem cell that is trapped centrally within the tumor mass should efficiently compete for space and resources required for clonal expansion^20, 21^. Based on our data, and supported by the results of our simulation model, which implemented position as the only factor determining tumor cell behavior, we therefore propose that competition of colon cancer cells for clonal expansion is mainly restricted to the leading tumor edge. The phenotype of tumor cells within expanding clones may still be variable, depending on the individual genetic background of the tumor, and may secondarily be influenced by a position-related tumor microenvironment^3, 5^.

Previous attempts to follow individually labeled tumor cells over time, independently of their phenotype, either used murine models, or lentiviral color- or bar-coding methods for random genetic labeling of tumor cells in vitro before xenotransplantation into mice^22–24^. While the approach in murine models is not applicable to human malignancies, the in vitro labeling approach has the caveat that clonal cell tracing cannot be induced after secondary tumor architectures have formed, and thus precludes access to clonal fate data of individual tumor cells. By combining the advantages of inducible clonal cell tracing and lentiviral delivery, we overcome these restrictions, and for the first time demonstrate a constant drift toward oligoclonality within colon cancer that appears to be based on clonal competition and axial outgrowth. However, although we simulate these dynamics in our simulation model by neutral stochastic competition of tumor cells at the leading tumor edge, the biological basis for clonal competition yet remains to be determined. Also, due to a limited number of different fluorescent colors, some aspects including the significance of neighboring clones with identical colors or clone fragmentation during outgrowth may be missed by our labeling strategy and require further study.

Importantly, in our model, clonal competition does not depend on mutational evolution. Although additionally acquired mutations in individual tumor cell subclones may provide fitness advantage, genetic changes that substantially alter the clonal composition of a final tumor are assumed to be rare events in rapidly expanding cell populations^25^. An inferred “Big Bang” model of colon cancer evolution therefore suggested that clonal dynamics in established tumors are mainly devoid of substantial mutational evolution^26^. In line with this idea, we found no differences in driver mutation profiles of individual tumor cell subclones. Therefore, we suggest that clonal competition in colon cancer is mainly determined by tumor cell position and may continuously occur throughout the lifespan of a tumor. Nevertheless, it remains to be determined to what extent other heritable traits may have an impact on clonal architecture and growth dynamics, since others reported epigenetic differences among subclones of colonic adenomas and colon cancers^27, 28^.

In contrast to unperturbed tumor growth, mutational evolution certainly plays an important role in acquired resistance to targeted therapy^29^. Previous data suggested that treatment protocols stabilizing tumor growth rather than attempting to eradicate the tumor may prolong cancer survival^30^. Our data suggesting continuous clonal competition may explain such findings. If treatment-resistant tumor subclones have to compete for space and resources with treatment-sensitive subclones that may prevail under gentle targeted therapy, loss of resistant clones due to necrosis in the tumor center may occur by chance. In contrast, harsher-targeted therapy may eliminate sensitive tumor cell clones and strongly favor quick outgrowth of resistant clones with earlier treatment failure. This hypothesis, however, will require further experimental proof, and may then inform the design of future-targeted therapeutic approaches for patients with colorectal cancer.

## Methods

### Tumor specimens, immunohistochemistry and immune fluorescence

Colon cancer specimens from patients that underwent surgical resection at the University of Munich (LMU) were drawn from the archives of the Institute of Pathology. Specimens were anonymized, and the need for consent was waived by the institutional ethics committee of the Medical Faculty of the LMU. Immunohistochemistry was done on 5 µm tissue sections of primary cancer specimens or xenograft tumors, as previously described^4^, using the antibodies listed in Supplementary Table 2. Stained slides were then inspected by light microscopy for the distribution of each marker antigen and categorized as negative, polarized if expression gradients from leading tumor edge to tumor center were observed, or diffuse if such gradients were absent. Ki67 proliferation was separately assessed at the leading tumor edge and in the tumor center. Relative BrdU staining intensity was quantified continuously from leading tumor edge to tumor center using ImageJ (NIH). For immune fluorescence, 5 µm sections of xenograft tumors were deparaffinized and antigens were retrieved in Target Retrieval Solution (Dako) for 20 min in a microwave oven. Sections were then incubated with primary antibodies listed in Supplementary Table 2. Secondary Alexa Fluor 405-, 488-, and 555-conjugated antibodies were used for visualization, and for some experiments nuclei were counterstained with DAPI (Vector Laboratories). Confocal fluorescence images were taken on a LSM 700 laser scanning microscope using the ZEN software (Zeiss).

### Lentiviral vectors

All template plasmids were obtained through Addgene (www.addgene.org). For the inducible pLenti TetO-CreERT2 expression vector, we amplified CreERT2 from pCAG-CreERT2 (a gift from Connie Cepko) by PCR, and inserted it between BamH1 and Xba1 restriction sites of pLenti CMVTRE3G eGFP Puro (a gift from Eric Campeau), replacing eGFP by CreERT2. For the Cre-sensitive recombination vector pLenti Multicolor, we first PCR-amplified expression cassettes for Kusabira orange, mCherry, and EYFP from CMV-Brainbow 1.1 M^30^, and EBFP2 from pEBFP2-Nuc (a gift from Robert Campbell), using primers that omitted membrane or nuclear localization signals, respectively. Amplicons then were inserted into EcoRV sites of pcDNA3.1 (Invitrogen), and the 3′ ends of mCherry, EYFP, and EBFP2 were replaced from BsrG1 to Not1 restriction sites by synthetic sequences that added FLAG, V5, or VSV tags, respectively. Kusabira orange and tagged fluorescent color coding genes were then sequentially inserted into a plasmid with synthetic paired loxN, lox2272, and loxP sites. The whole expression cassette was then inserted between Age1 and Sal1 sites of pLenti PGK-GFP (a gift from Didier Trono), replacing GFP. Finally, the PGK promoter was replaced by an EF1α promoter, yielding pLenti Multicolor. Vector elements were verified by restriction analysis and sequencing.

### Cell culture and lentiviral transductions

HEK293 and HCT116 cells were obtained from ATCC and SW1222 from the Ludwig Institute for Cancer Research (New York, USA). Cell lines were authenticated using short-tandem repeat profiling, tested negative for mycoplasma contamination and cultured in Dulbecco’s modified Eagle medium (DMEM) supplemented with 10% FBS, 100 U/ml penicillin, and 0.1 mg/ml streptomycin (Biochrom). For transductions, lentivirus was produced in HEK293 cells by co-transfection with lentiviral vector, pCMV-dR8.91, and pMD2.G, as previously described^8^. Virus containing medium was passed through 0.45 µm filters (Millipore), mixed 1:1 with DMEM, and used to infect HCT116 and SW1222 colon cancer cells in the presence of 8 µg/ml polybrene (Sigma-Aldrich). pLenti rtTA3G, pLenti TetO-CreERT2, and pLenti Multicolor triple transduced cells were then single cell subcloned by limiting dilution, expanded, and tested for recombination in vitro by addition of 1 µg/ml doxycycline and 10 µM 4-hydroxytamoxifen (Sigma-Aldrich), before xenotransplantation into mice.

### Tumor xenografts

Mouse experiments were reviewed and approved by the Regierung von Oberbayern. A total of 1 × 10^6^ single clone-expanded SW1222 or HCT116 colon cancer cells carrying the multicolor lineage tracing constructs were suspended in 100 μl of a 1:1 mixture of PBS and growth factor-depleted Matrigel (Corning), and injected subcutaneously into 6–8-week-old male NOD/SCID mice (NOD.CB17-Prkdcscid, The Jackson Laboratory) for xenograft formation. When tumor diameters reached 7 mm, recombination of pLenti Multicolor transgenes was induced by 1 mg doxycycline p.o. for 3 consecutive days, followed by 3 mg tamoxifen i.p. (Sigma-Aldrich). For BrdU tracing, mice were injected once with a 1.25 mg BrdU pulse. At distinct time points, mice were killed, tumors were removed, formalin fixed, and paraffin embedded for further analyses.

### Panel sequencing

For next-generation panel sequencing, we used the Ion AmpliSeq Cancer Hotspot Panel v2, covering the mutational status of 50 oncogenes and tumor suppressor genes, according to the manufacturer’s protocol (Life Technologies). Thirty-one days after recombination, individual clones from immunohistochemically stained slides of different SW1222 and HCT116 xenograft tumors were microdissected and 1–5 ng DNA were used as template for library construction. Multiplexed libraries were then sequenced on an Ion Personal Genome Machine (Thermo Fisher). Reads were mapped to human reference genome hg19 and filtered for non-synonymous variants.

### Analysis of clone characteristics

To determine clone sizes, we counted neighboring tumor cells with identical fluorescent colors on confocal images. For each clone, we then determined the positions of each cell C (*x*_C_, *y*_C_), as well as the closest positions of leading tumor edge E (*x*_E_, *y*_E_) and tumor necrosis N (*x*_N_, *y*_N_) using ImageJ (NIH). By geometric shifting and rotation, we then transformed coordinates so that E′ (0, 0) and N′ (*x*_N_′, *y*_N_′) with *x*_N_′ = *y*_N_′. The resulting cell positions C′ (*x*_C_′, *y*_C_′) for each clone were then analyzed for linear correlation by *t*-test, the slope of the linear regression *m* was determined, and the angle *θ* of the line of best fit with the *x*-axis was calculated by *θ* = tan^−1^(*m*). The angle *α* of each clone relative to a tangent to the leading tumor edge then resulted from *α* = *θ* + 45°.

### Simulation model and code availability

The two-dimensional spatial simulation model for clonal dynamics was implemented in VBA-Excel (code available in Supplementary Data 1). In a worksheet “Clones”, simulating 60 × 60 cells, random numbers from 1 to 3600 were distributed. These are illustrated in a 60 × 60 matrix in worksheet “Graphics” with 10 different colors, determined by clone number modulo 10. For each simulation cycle for cells at the bottom row, representing the leading tumor edge, each cell content is either copied to the neighboring cell on the left or right, simulating lateral expansion for clonal competition, or to the cell above, simulating clonal outgrowth toward the tumor center. This behavior is determined at random. For all remaining cells, contents are copied to the cell above, while this is restricted to every Nth row, with *N* simulating the proliferation gradient from leading tumor edge (bottom row) to tumor center (other rows). Contents of cells that are to be replaced are shifted to cells immediately above, causing loss of clones only at the top row of the model, which simulates loss into tumor necrosis. Frequencies are recorded in worksheet “Numbers” and represented in a diagram in worksheet “Graphics”.

### Statistical analysis

Sample sizes were based on preliminary data and previous experience. Xenograft bearing mice were randomly assigned to groups with different observation time points after multicolor recombination, and no xenografts were excluded from analysis. Investigators were not blinded to group allocations. Appropriate statistical tests were used to compare data with similar variances and are referenced in figure legends. Biological replicates are given as *n* values. All graphs show mean and error bars represent standard deviation (s.d.) or standard error of the mean (s.e.m.). Differences were considered statistically significant when *P* < 0.05, and *P* values are given within figures or figure legends. Statistics were calculated with GraphPad Prism.

### Data availability

Data from genetic mutation analyses are given in Supplementary Table 1. All other data are available from the corresponding author on reasonable request.

## Electronic supplementary material

Supplementary Information

Description of Additional Supplementary Files

Supplementary Data 1
