# Supplementary material for: Multicolor lineage tracing reveals clonal architecture and dynamics in colon cancer
Source: Nat Commun. 2017 Nov 10;8:1406. doi: 10.1038/s41467-017-00976-9 (PMC5681634; doi:10.1038/s41467-017-00976-9)
Supplement: Supplementary file 1 — Supplementary Information [file 41467_2017_976_MOESM1_ESM.pdf]

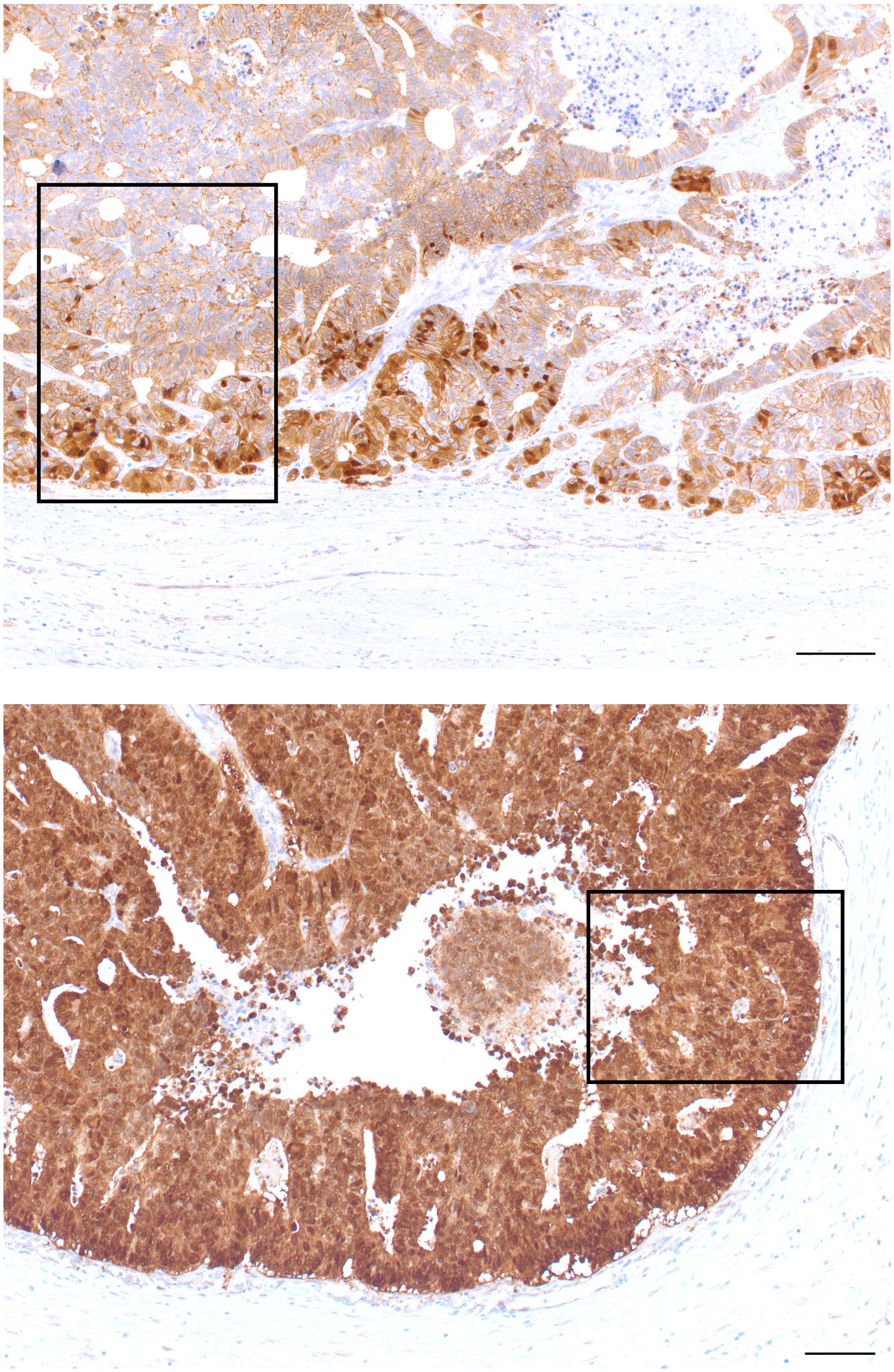

Supplementary Figure 1. Overview micrographs of primary colon cancers. Immunohistochemistry in representative primary colon cancers showing polarized (*upper panel*) or diffuse (*lower panel*) distribution of nuclear β-catenin. *Boxes* indicate areas that are shown in Figure 1. *Scale bars*, 100 μm.

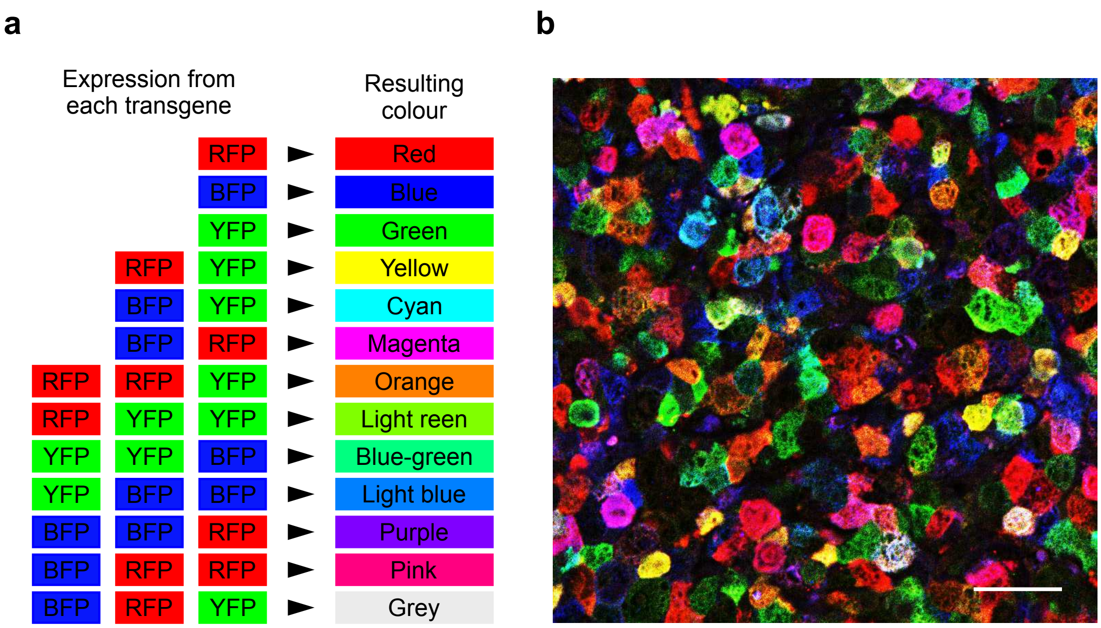

Supplementary Figure 2. Mixed fluorescent color expression due to multiple vector insertions. (**a**) Upon insertions of e.g. three copies of pLenti Multicolor, by chance one, two or three inserted transgenes may recombine, resulting in up to 13 different single or mixed fluorescent colors. (**b**) Fluorescent color spectrum in an HCT116 xenograft tumor three days after recombination. *Scale bar*, 25 μm.

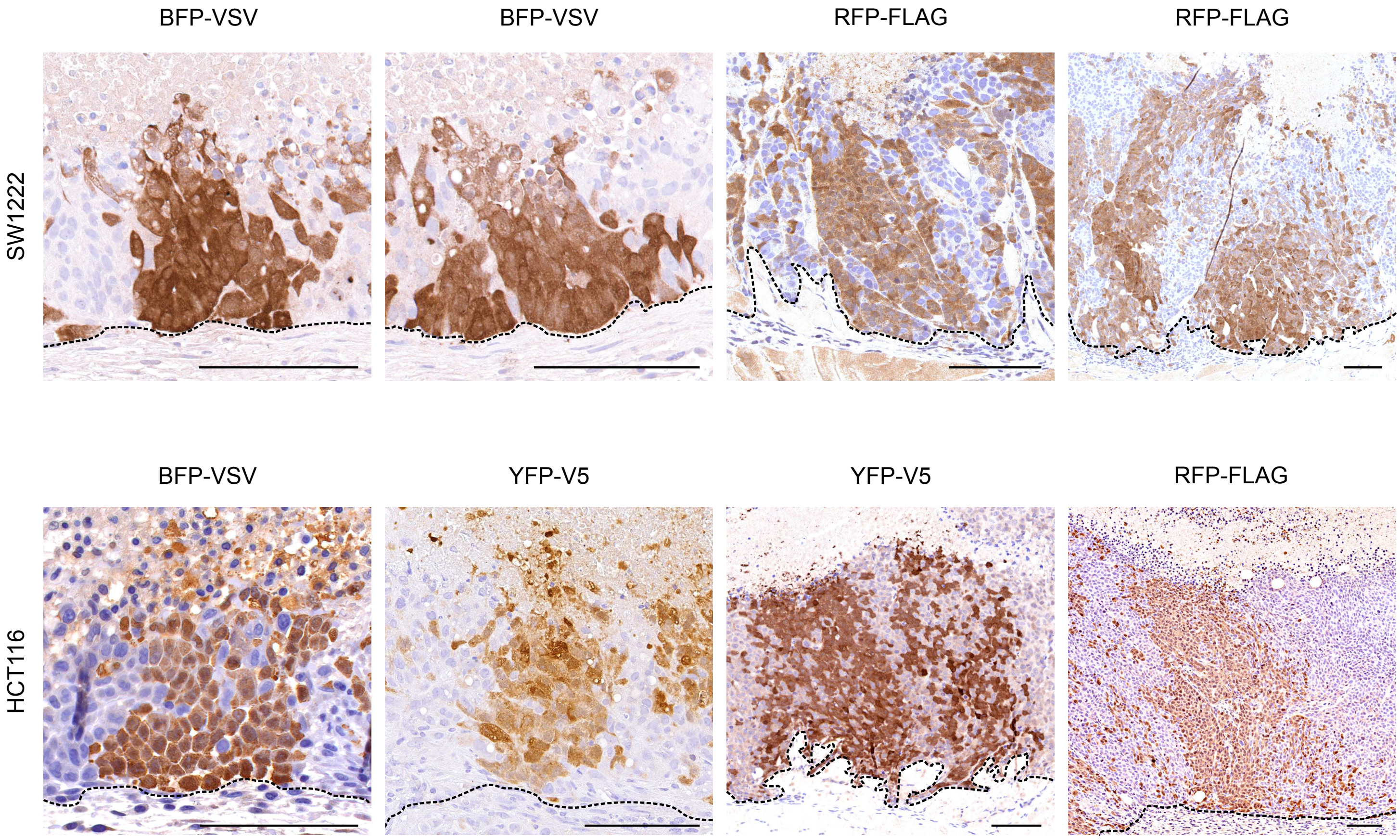

Supplementary Figure 3. Phenotypes of subclones in colon cancer xenografts. Immunohistochemistry for indicated tagged fluorescence proteins illustrates representative clonal patches in SW1222 and HCT116 xenograft tumors 31 days after recombination. Micrographs show tumors from leading tumor edge (indicated by *dotted lines* at *image bottom*) to tumor center or central tumor necrosis (*image top*). *Scale bars*, 100 μm.

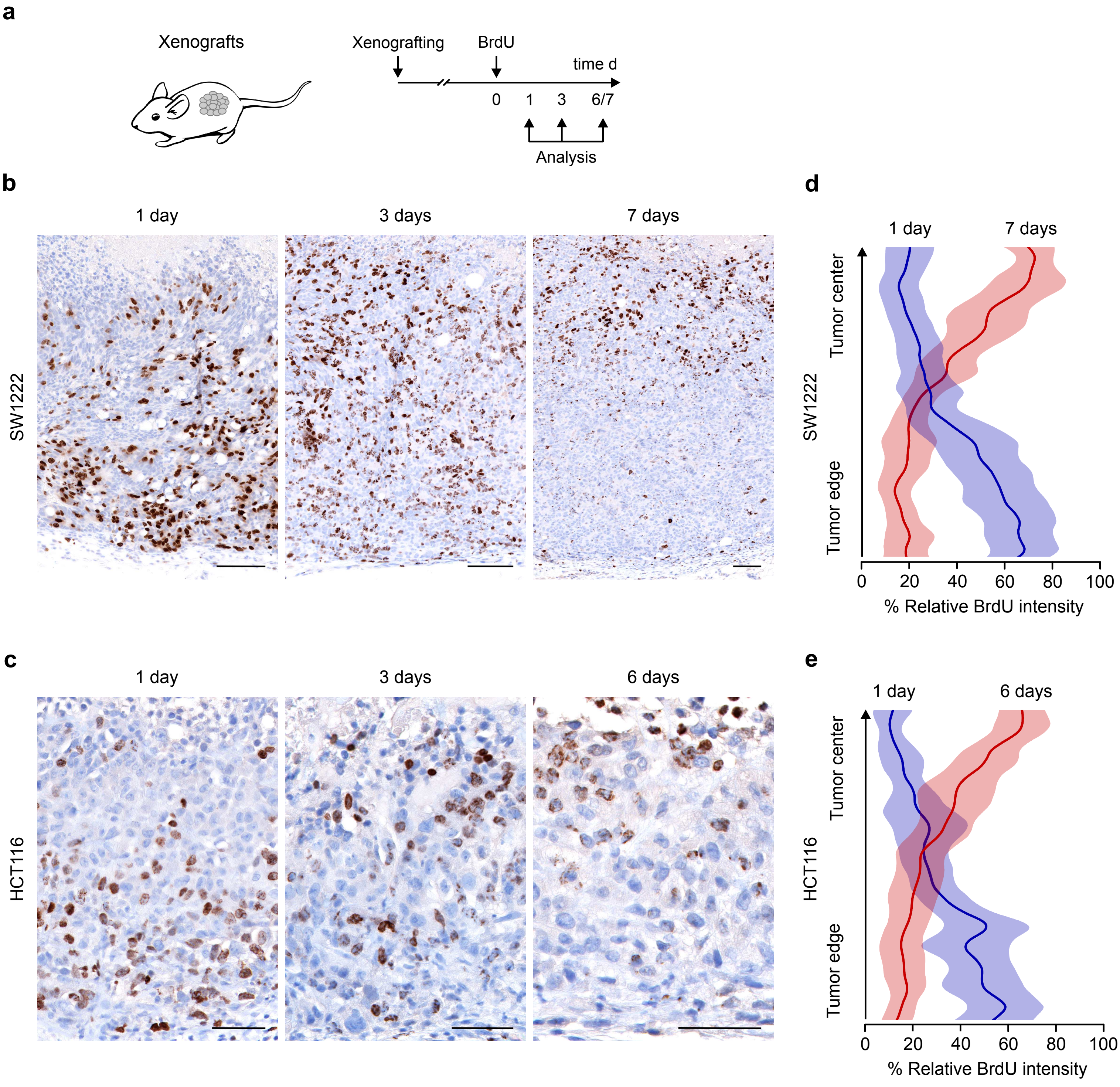

Supplementary Figure 4. BrdU tracing in colon cancer xenografts. (**a**) Schema and experimental schedule. (**b, c**) Immunohistochemistry for BrdU in SW1222 and HCT116 xenograft tumors at indicated time points after BrdU pulse. Micrographs show tumors from leading tumor edge (*image bottom*) to tumor center or central tumor necrosis (*image top*). *Scale bars*, 100 µm in (**b**) and 50 µm in (**c**). (**d, e**) Quantification of average relative BrdU staining intensity from tumor edge to tumor center. Data are mean with 95 % confidence bands and derived from different xenograft areas ($n \geq 20$) of independent biological replicates ($n \geq 3$).

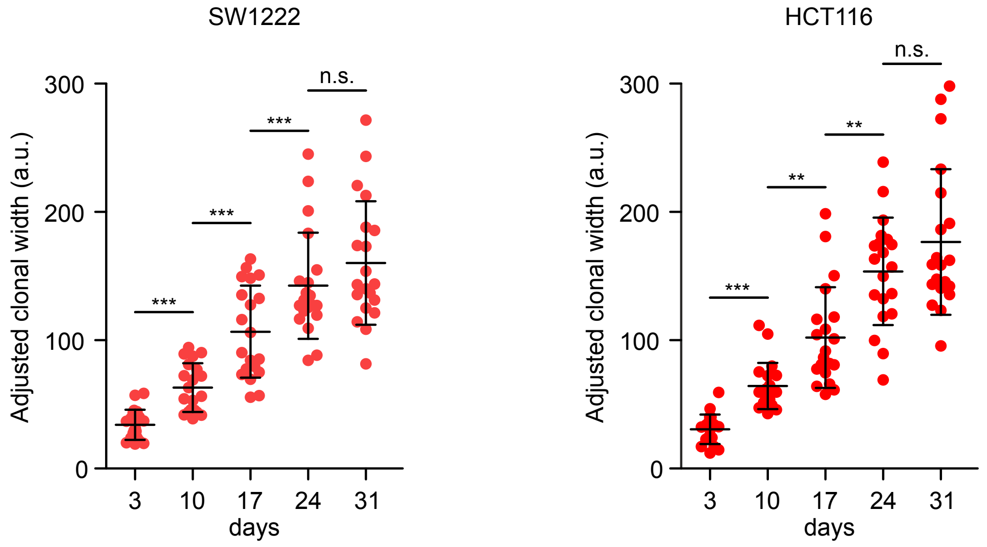

Supplementary Figure 5. Lateral clonal expansion in colon cancer xenografts. Measurements of clonal width at the leading tumor edge divided by relative changes in tumor circumference (adjusted clonal width) are shown for indicated time points after multicolor recombination. a.u., arbitrary units. *Error bars* indicate mean ± s.d. from $n = 20$ clones per indicated time point. **, $P < 0.01$; ***, $P < 0.001$; n.s., nonsignificant by two-sided *t* test.

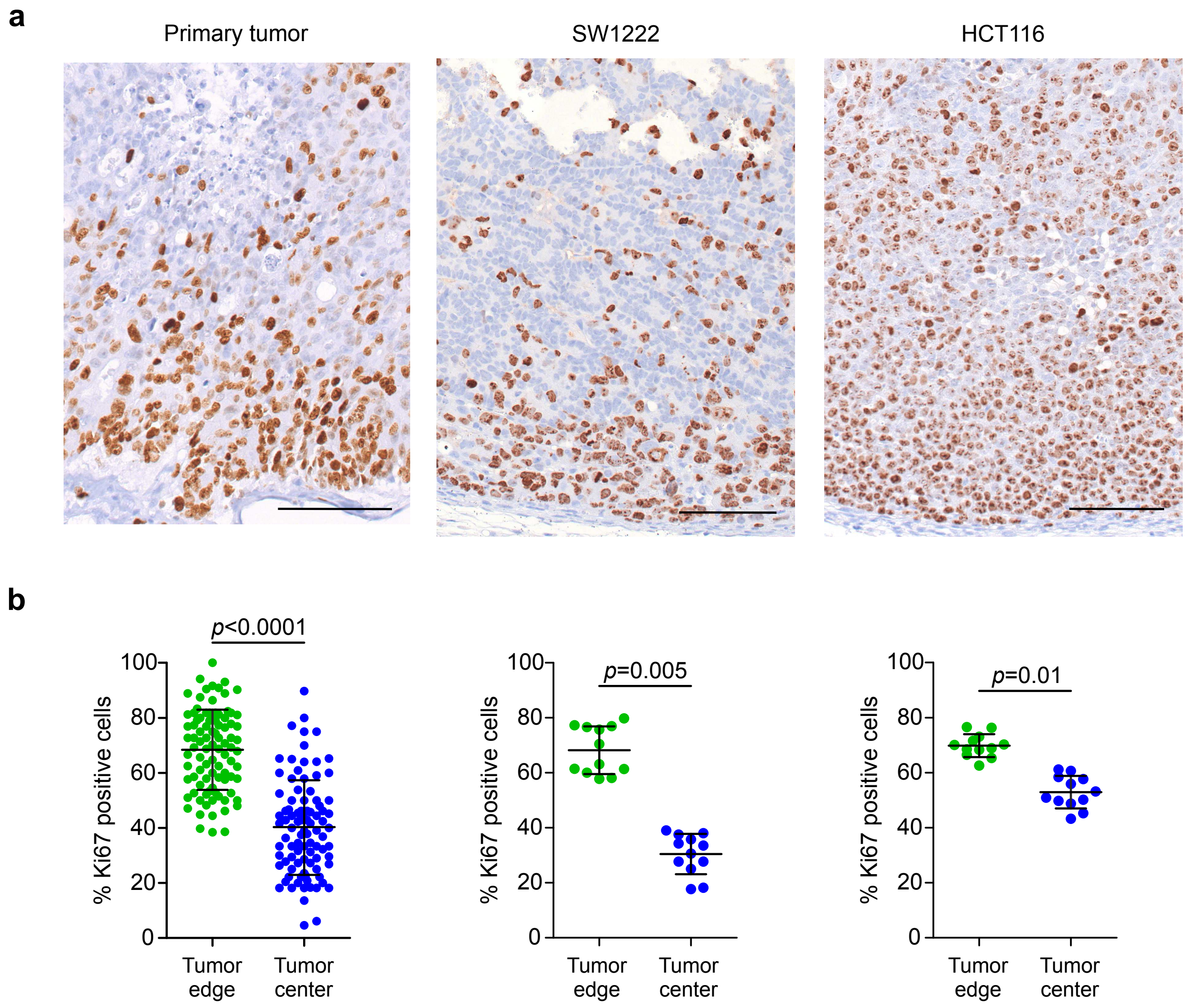

Supplementary Figure 6. Proliferation gradients in colon cancer. (**a**) Representative immunohistochemistry and (**b**) quantification of Ki67 in primary colon cancers (n = 92) and in SW1222 and HCT116 xenograft tumors (4 different areas in 3 biological replicates), as indicated. Micrographs show tumors from leading tumor edge (*image bottom*) to tumor center or central tumor necrosis (*image top*). *Scale bars*, 100 µm. *Error bars* indicate mean ± s.d. *P* values are two-sided *t* test results.

Supplementary Table 1: Mutational status of individual microdissected subclones of SW1222 and HCT116 xenograft tumors, 31 days after recombination.

		SW1222 clones					HCT116 clones				
**Gene**	**Variant**	**#1**	**#2**	**#3**	**#4**	**#5**	**#1**	**#2**	**#3**	**#4**	**#5**
APC	G1306T	+	+	+	+	+					
KRAS	A146V	+	+	+	+	+					
CTNNB1	S45del						+	+	+	+	+
KRAS	G13D						+	+	+	+	+
PIK3CA	H1047R						+	+	+	+	+
SMO	V404M						+	+	+	+	+
ABL1	D276G						+	+	+	+	+

Supplementary Table 2: Antibodies used for immunohistochemistry (IHC) and immunofluorescence (IF).

**Antibody**	**Species**	**Manufacturer**	**IHC**	**IF**
Alexa Flour 405 anti-mouse (ab175658)	Donkey	Abcam		1:500
Alexa Flour 488 anti-goat (A-11055)	Donkey	Invitrogen		1:500
Alexa Flour 568 anti-rabbit (ab175470)	Donkey	Abcam		1:500
β-catenin (610153)	Mouse	BD Biosciences	1:200	1:200
BrdU (sc-32323)	Mouse	Santa Cruz	1:100	
CK20 (sc-17113)	Goat	Santa Cruz	1:200	1:200
FLAG M2 (#2368)	Rabbit	Cell Signaling	1:500	1:300
FRA1 (sc-28310)	Mouse	Santa Cruz	1:100	1:50
GLUT1 (sc-377228)	Mouse	Santa Cruz	1:100	1:100
Ki67 (#9027)	Rabbit	Cell Signaling	1:200	
VSV-G (sc-66180)	Mouse	Santa Cruz	1:200	1:150
V5 (ab95038)	Goat	Abcam	1:400	1:300
