# Supplementary material for: Multicolor lineage tracing reveals clonal architecture and dynamics in colon cancer
Source: Nat Commun. 2017 Nov 10;8:1406. doi: 10.1038/s41467-017-00976-9 (PMC5681634; doi:10.1038/s41467-017-00976-9)
Supplement: Supplementary file 2 — Description of Additional Supplementary Files [file 41467_2017_976_MOESM2_ESM.pdf]

Description of Additional Supplementary Files

File Name: Supplementary Data 1
Description: Simulation of clonal dynamics in colon cancer.
